# A Novel Malate Dehydrogenase 2 Inhibitor Suppresses Hypoxia-Inducible Factor-1 by Regulating Mitochondrial Respiration

**DOI:** 10.1371/journal.pone.0162568

**Published:** 2016-09-09

**Authors:** Hyun Seung Ban, Xuezhen Xu, Kusik Jang, Inhyub Kim, Bo-Kyung Kim, Kyeong Lee, Misun Won

**Affiliations:** 1 Metabolic Regulation Research Center, Korea Research Institute of Bioscience and Biotechnology, Daejeon, Korea; 2 Biomolecular Science, University of Science and Technology, Daejeon, Korea; 3 College of Pharmacy, Dongguk University-Seoul, Goyang, Korea; 4 Personalized Genomic Medicine Research Center, Korea Research Institute of Bioscience and Biotechnology, Daejeon, Korea; 5 Functional Genomics, University of Science and Technology, Daejeon, Korea; Seoul National University College of Pharmacy, REPUBLIC OF KOREA

## Abstract

We previously reported that hypoxia-inducible factor (HIF)-1 inhibitor LW6, an aryloxyacetylamino benzoic acid derivative, inhibits malate dehydrogenase 2 (MDH2) activity during the mitochondrial tricarboxylic acid (TCA) cycle. In this study, we present a novel MDH2 inhibitor compound 7 containing benzohydrazide moiety, which was identified through structure-based virtual screening of chemical library. Similar to LW6, compound 7 inhibited MDH2 activity in a competitive fashion, thereby reducing NADH level. Consequently, compound 7 reduced oxygen consumption and ATP production during the mitochondrial respiration cycle, resulting in increased intracellular oxygen concentration. Therefore, compound 7 suppressed the accumulation of HIF-1α and expression of its target genes, vascular endothelial growth factor (VEGF) and glucose transporter 1 (GLUT1). Moreover, reduction in ATP content activated AMPK, thereby inactivating ACC and mTOR the downstream pathways. As expected, compound 7 exhibited significant growth inhibition of human colorectal cancer HCT116 cells. Compound 7 demonstrated substantial anti-tumor efficacy in an *in vivo* xenograft assay using HCT116 mouse model. Taken together, a novel MDH2 inhibitor, compound 7, suppressed HIF-1α accumulation via reduction of oxygen consumption and ATP production, integrating metabolism into anti-cancer efficacy in cancer cells.

## Introduction

Cancer cells possess abnormal metabolic properties, such as aerobic glycolysis, high fatty acid synthesis, and rapid glutamine metabolism [1,2]. These metabolic alterations are associated with cancer progression and therapeutic resistance to cancer treatment [3,4]. Adaptation of cancer cells to hypoxia leads to the alteration of metabolism. A hypoxia-responsive transcription factor, hypoxia-inducible factor (HIF)-1α, has been implicated in the regulation of tumor angiogenesis, metastasis and proliferation in response to hypoxia [5–7]. HIF-1α is correlated with poor cancer prognosis and chemotherapeutic resistance in various cancers [8], and many HIF-1α inhibitors have been studied as potential anticancer agents [9,10].

Previously, we developed HIF-1α inhibitor LW6 from an aryloxyacetylamino benzoic acid scaffold [11–13] and showed that LW6 inhibits accumulation of HIF-1α and expression of its target genes in colorectal cancer cells. Using multifunctional chemical probes of LW6, we determined that it directly targets malate dehydrogenase 2 (MDH2) [14,15], which is involved in the regulation of HIF-1α accumulation under hypoxia.

As a mitochondrial protein, MDH2 is a key enzyme in the tricarboxylic acid cycle that catalyzes the interconversion of malate and oxaloacetate by utilizing the NAD/NADH coenzyme system [16]. Recently, MDH2 has been recognized as an attractive target for cancer treatment. The elevation of MDH2 expression has been implicated in prostate cancer resistance to docetaxel-chemotherapy [17]. Moreover, overexpression of MDH2 in clinical prostate cancer has resulted in shortened periods of relapse-free survival after exposure to chemotherapy [17]. In addition, MDH2 is involved in doxorubicin resistance in uterine cancer cells [18].

To search for a novel MDH2 inhibitor as a potential anticancer agent, we performed virtual screening of a compound library based on the structure of LW6 and identified a benzohydrazide derivative compound 7. We studied the kinetics of binding between MDH2 and compound 7, as well as the mechanism of compound 7 in the inhibition of HIF-1α accumulation. In addition, an assessment of the *in vivo* anti-tumor efficacy of compound 7 was carried out in a xenograft mouse model.

## Materials and Methods

### Chemicals

All compounds used in this study were purchased from Ambinter (Paris, France). Stock solutions of compounds were prepared in DMSO at 10 mM and stored at -20°C.

### Cell lines and cell culture

The human colorectal cancer HCT116, cervical carcinoma HeLa, hepatocellular carcinoma HepG2, and non-small cell lung cancer H1703 cells were obtained from the KRIBB cell line bank (Daejeon, Korea). HCT116 cells were cultured in a 5% CO_2_ atmosphere at 37°C in Dulbecco’s modified Eagle’s medium (Gibco, Carlsbad, CA, USA) supplemented with 5% fetal bovine serum (Gibco), 100 U/ml penicillin, and 100 μg/ml streptomycin (Gibco). Cells were seeded at a density of 5 × 10^5^ cells/ml/well in a 12-well tissue culture plate at 37°C for 20 h prior to subsequent experiments. Hypoxic conditions were achieved by incubating the cells in 1% O_2_, 94% N_2_, and 5% CO_2_ in a multigas incubator (Sanyo, Osaka, Japan).

### MDH2 activity assay

MDH2 enzyme activity was determined by oxaloacetate-dependent NADH oxidation assays as previously described [15]. The reaction was performed in 100 mM potassium phosphate buffer (pH 7.4) with 0.25 nM rhMDH2, 200 μM oxaloacetic acid, and 200 μM NADH. In the case of the kinetic assay, the reaction was performed with 0.25 nM rhMDH2, 600 μM oxaloacetic acid, and various concentrations of NADH (60, 75, 100, 150, and 300 μM). The NADH concentration was determined by measuring absorbance at 340 nm. The Vmax and Km were determined from double-reciprocal Lineweaver-Burk plots using Sigmaplot 13.0 (Systat Software), and the velocity was plotted against the concentration of NADH.

### Virtual screening

Virtual screening was conducted by SYBYL-X software’s (version 2.1.1) by implementing a topomer search model for the whole molecule using the UNITY search function. In the topomer search, a 3D structure of LW6 was used as the template, and the ligand source included all purchasable compounds, shard compounds, all boutique compounds and boutique shard compounds in the zinc database. From the database, 156 ligands were removed by file format translation, and 919,548 compounds were removed by duplication. After removal, 28,604,480 compounds remained. Furthermore, CONCORD was chosen in Query and Database for input options and data preparation, and other parameters were determined following the default settings. The protein-LW6 complex was used in the UNITY search, and we defined 4 queries depending on the interaction of LW6 with protein. The result of the topomer search was used in a database for the unity search. The selected 16 compounds were purchased from Ambinter (http://www.ambinter.com).

### Molecular docking study

A molecular docking study was carried out using Surflex Dock interfaced with the SYBYL-X software (version 2.1.1). The energy minimization used for molecule docking was performed using Tripos Force Field, and the Gasteigere-Huckel charge was applied with 1,000,000,000 max iterations of the Powell gradient method with a convergence criterion of 0.05 kcal/mol. To prepare the protein, all hydrogens and AMBER7 FF99 charges were added, and the side-chain amides were fixed. A staged minimization was performed using Tripos Force Field and the AMBER7 FF99 charge with 1,000 iterations of the Powell method with a convergence criterion of 0.5 kcal/mol at none of the initial optimizations. The default settings were accepted for all other parameters. The reference protein applied to the docking system was taken from the crystal structure of Human Malate Dehydrogenase Type 2 (MDH2, PDB code: 2DFD) chain A. The binding mode was calculated using the Surflex-Dock score (Total_Score, Crash, and Polar). In addition, the D score, PMF score, G score, and ChemScore were also calculated.

### Measurement of mitochondrial respiration

The oxygen consumption rate (OCR) in cells was measured using the XF24 extracellular flux analyzer (Seahorse Biosciences, North Billerica, MA), as previously described [14]. HCT116 cells in XF24 cell culture plates were incubated for 24 h (Seahorse Biosciences) and incubated for an additional 30 min at 37°C without CO_2_ in XF assay media (pH 7.4, Seahorse Biosciences) containing various concentrations of compound 7. After determining the basal OCR, oligomycin (1 μM), carbonylcyanide *p*-trifluoromethoxyphenylhydrazone (0.5 μM), and rotenone (1 μM)/antimycin A (1 μM) were added sequentially, and the OCR was determined following each addition.

### Determination of ATP content

Intracellular ATP content was determined by an ENLITEN ATP Assay System (Promega, Madison, WI, USA) according to the manufacturer’s instructions. HCT116 cells (5 × 10^4^ cells/96 well) were incubated for 6 h with or without drugs. After removing the supernatant, ATP was extracted with 1% trichloroacetic acid, and the solution was neutralized with Tris buffer (pH 7.8). After addition of rL/L reagent, ATP content was measured using a Victor™ X Light luminescence reader (Perkin Elmer, Boston, MA, USA) [14].

### Immunoblot analysis

Cells were lysed with RIPA buffer (20 mM HEPES, pH 7.4, 1% Triton X-100, 10% glycerol, 1 mM EDTA, 5 mM sodium fluoride, 10 μg/mL phenylmethylsulphonylfluoride, and 1 mM sodium vanadate) for 15 min at 4°C and centrifuged at 13,000 rpm for 15 min. Lysates were subsequently boiled for 5 min in 5X sample buffer (50 mM Tris, pH 7.4, 4% sodium dodecyl sulfate (SDS), 10% glycerol, 4% 2-thioethanol, and 50 μg/mL Bromophenol blue) at a ratio of 4:1. Protein samples were subjected to SDS-polyacrylamide gel electrophoresis (PAGE), transferred to PVDF membranes (Millipore, Billerica, MA), and immunoblotted with the following antibodies: HIF-1α (#610958, BD Transduction Laboratories, San Diego, CA, USA), MDH2 (YF-MA14165, Abfrontier, Seoul, Korea), P-ACC (#3661, Cell signaling, Danvers, MA, USA), ACC (#3676, Cell signaling), P-AMPK (#2535, Cell signaling), AMPK (#5831, Cell signaling), and β-actin (#8227, Abcam, Cambridge, UK). Protein expression was visualized on Kodak Biomax X-ray film (Kodak, Rochester, NY, USA).

### Reporter gene assay

HCT116 cells stably expressing hypoxia response element (HRE)-dependent firefly luciferase reporter construct (HRE-Luc) and a CMV-Renilla luciferase reporter construct were established using the Cignal™ Lenti Reporter assay system (SABiosciences, Frederick, MD, USA) according to the manufacturer’s instructions. Cells were incubated for 12 h in the presence or absence of drugs under normoxic or hypoxic condition. After removing the supernatant, luciferase activity was measured using a Dual-Luciferase Assay System (Promega, Madison, WI, USA), according to the manufacturer’s instructions, with a Victor™ X Light luminescence reader [15].

### RT-PCR

Total RNA was isolated using Trizol reagent (Invitrogen, Carlsbad, CA, USA) according to the manufacturer’s instructions. One μg of total RNA was reverse transcribed using TOPscript RT DryMIX kit (Enzynomics, Seoul, Korea). The PCR primers used were 5’-GCAGCCAGATCTCGGCGAAG-3’ (forward) and 5’-CTGTGTCCAGTTAGTTCAAA-3’ (reverse) for HIF-1α, 5’-GGTGGACATCTTCCAGGAGT-3’ (forward) and 5’-GGCTTGTCACATCTGCAAGT-3’ (reverse) for VEGF, 5’-ATGATTGGCTCCTTCTCTGT-3’ (forward) and 5’-TCAGCATCTCAAAGGACTTG-3’ (reverse) for GLUT1, and 5’-CATCGTGGCTAAACAGGTAC-3’ (forward) and 5’-GCACGACCTTGAGGGCAGC-3’ (reverse) for RPL13A. The amplified products were subjected to electrophoresis on 2% agarose gels and visualized by ethidium bromide staining.

### Detection of oxygen tension

Intracellular oxygen tension was detected using a hypoxia-detecting probe mono azo rhodamine (MAR, Goryo Chemical, Japan) as described [19]. HCT116 cells were incubated in the presence of drug and 500 nM MAR for 6 h under hypoxia. Then the fluorescence intensity was analyzed by IncuCyte live cell imaging system (Essen BioScience, Ann Arbor, MI, USA).

### Nile-red staining

Intracellular fatty acids were stained with Nile-red as described [20]. HCT116 cells incubated in the presence or absence of drug for 48 h were stained with 500 nM Nile-red fluorescent dye (Sigma-Aldrich, St. Louis, MO, USA) in PBS. The fluorescence stained cells were analyzed by an IncuCyte live cell imaging system (Essen BioScience, Ann Arbor, MI, USA).

### Xenograft model

The *in vivo* antitumor activity of MDH2 inhibitor was determined by performing a HCT116 xenograft mouse experiment. All animal experimental protocols were approved by the bioethics committee of the Korea Research Institute of Bioscience and Biotechnology. Six-week-old female nude mice were subcutaneously inoculated with 5 × 10^6^ HCT116 cells in the right flank. When the tumor volume reached 80–100 mm^3^, compound 7 (10 and 20 mg/kg) was administered intraperitoneally once a day for 14 days. Tumor volume was measured every other day with calipers and calculated using following equation: V (mm^3^) = (Length × Width × Height) × 0.5. At the end of the experiment, mice were sacrificed by CO_2_ inhalation.

### Statistical analysis

The statistical significance of the results was analyzed using Student’s t-tests for unpaired observations and Dunnett’s test for multiple comparisons.

## Results

### Virtual screening

To identify novel inhibitors of MDH2, a step-wise strategy for virtual screening was employed using topomer search and UNITY search. In topomer search, we found many ligands that exhibited similarities with LW6 from the zinc database. Then, we used the unity search function to screen again to reduce ligand numbers. After the two-step screening, we obtained 14,252 compounds that were more similar to LW6. Further, we performed additional screening based on QFIT score and NUMHITS score. Next, the 3D structures of the compounds were compared with that of LW6 to assess whether they had high similarity and potential drug properties. Finally we selected 16 ligands and purchased them from the chemical vendor.

### Compound 7 inhibits MDH2 activity

**Fig 1:**
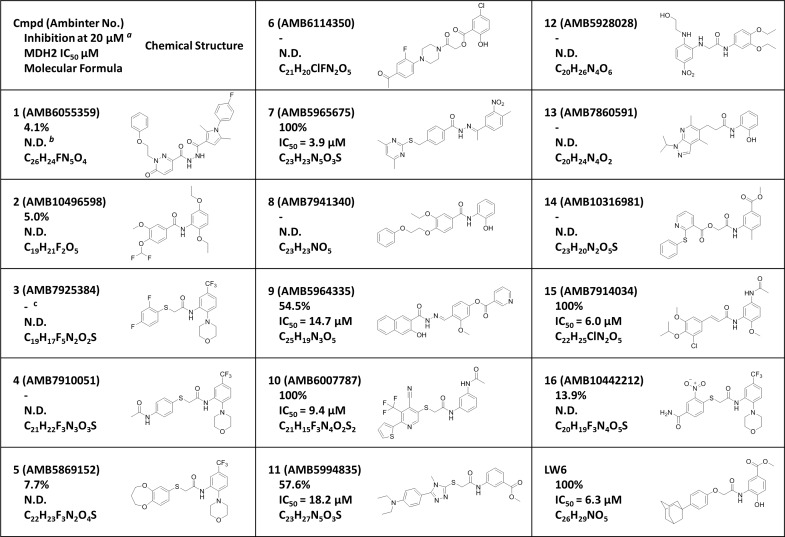
*In vitro* MDH2 inhibitory activity of compounds. ^*a*^MDH2 enzyme activity was determined by oxaloacetate-dependent NADH oxidation assays. Values are the means of three experiments. ^*b*^N.D. measns non-determined. ^*c*^‘–’ means no effect.

**Fig 2:**
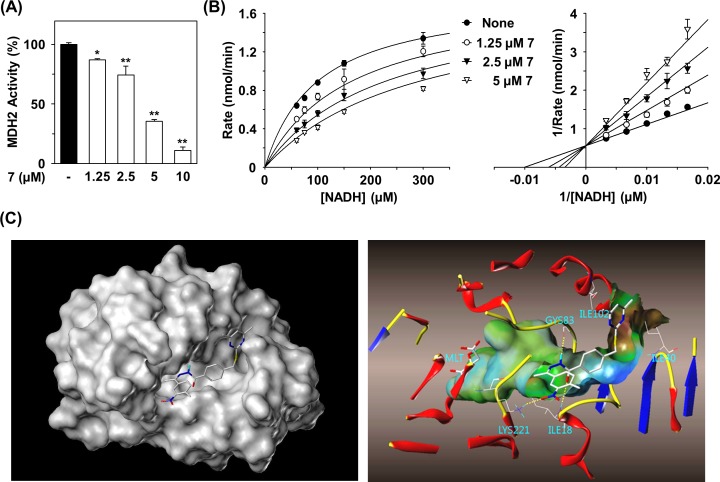
Inhibition of MDH2 activity by compound 7. (A) Effect of compound 7 on MDH2 activity. *In vitro* MDH2 activity was determined by oxaloacetate-dependent NADH oxidation assays. Statistical significance: *P < 0.05 and **P < 0.01, compared with untreated control. (B) Kinetic study of MDH2 by compound 7. Double-reciprocal plot of the inhibitory effect of compound 7 against NADH-dependent MDH2 activity. Concentration of compound 7 (in μM): 0 (closed circle), 1.25 (open circle), 2.5 (closed triangle), and 5 (open triangle). (C) Molecular docking study of MDH2 compound 7. Docking simulation showing the binding of compound 7 in the NADH binding site of MDH2 (PDB ID code 2DFD). The numbers of amino acid residues are based on PDB structure.

A total of 16 compounds identified by virtual screening of the compound library (Ambinter vender) were purchased (Fig 1). We first examined the effects of the 16 compounds on the MDH2 activity by determining the degree of oxaloacetate-dependent NADH oxidation. Compounds 7, 10, and 15 completely inhibited the activity of MDH2 at 20 μM, while compounds 9 and 11 moderately inhibited MDH2 activity. However, other compounds did not cause inhibition of MDH2 activity. Compound 7, (E)-4-((4,6-dimethylpyrimidin-2-ylthio)methyl)-N'-(1-(4-methyl-3-nitrophenyl)ethylidene)benzohydrazide, exerted the most potent inhibition of MDH2 with an IC_50_ of 3.9 μM (Fig 1 and Fig 2A), which was somewhat improved compared with that of LW6 (6.3 μM). The potency of compound 15, (E)-N-(5-acetamido-2-methoxyphenyl)-3-(3-chloro-4-isopropoxy-5-methoxyphenyl)acrylamide, on MDH2 inhibition was similar to that of LW6. To understand the inhibitory mechanism of compound 7 on MDH2 activity, an enzyme kinetics study was performed at various NADH concentrations. Double-reciprocal plots of enzyme kinetics revealed that compound 7 inhibited MDH2 activity in a competitive fashion with a Ki value of 2.3 μM (Fig 2B).

In order to investigate the binding mode of compound 7 with benzohydrazide scaffold, docking simulation was performed using the Surflex Dock software program based on the crystal structure of MDH2 (PDB code: 2DFD). Compound 7 was docked to the active site of MDH2 in place of the ligand nicotinamide-adenine-dinucleotide (NAD). As shown in Fig 2C, the nitro group of compound 7 interacted with the side chain of Lys223 by hydrogen bonding. Additionally, the carbonyl group and hydrazide group of the benzohydrazide portion of compound 7 made hydrogen-bonds with the backbone of Ile18 and Gly83, respectively. The dimethylpyrimidine group had hydrophobic interactions with sidechain of Ile40 and Ile102. Likewise, the 3D model suggests that the structure fits nicely into the pocket of MDH2 and, therefore, should have a very good overall score for docking. Furthermore, other scores such as the Crash score, Polar score, D score, PMF score, G score, and ChemScore also demonstrated that compound 7 is an inhibitor of MDH2.

### Compound 7 inhibits mitochondrial respiration by inhibiting MDH2 activity

**Fig 3:**
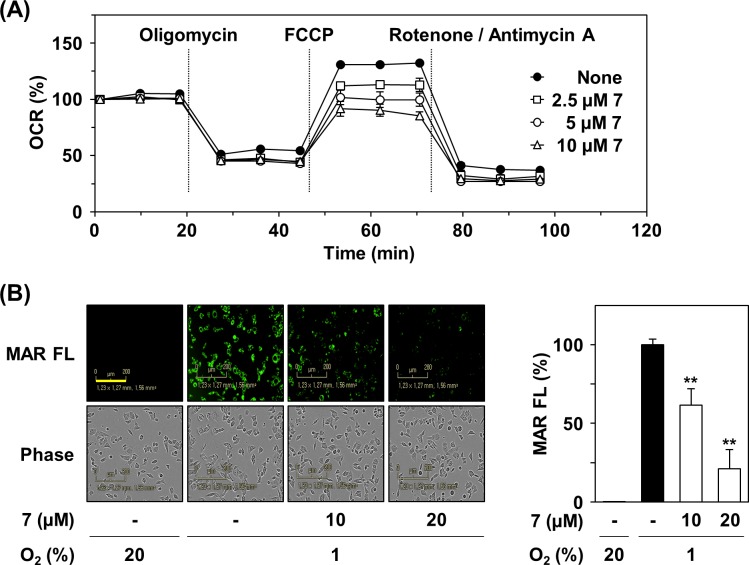
Inhibition of mitochondrial respiration by compound 7. (A) Effect of compound 7 on mitochondrial respiration. OCR was measured using XF24 extracellular flux analyzer by adding oligomycin (1 μM), FCCP (0.5 μM), and rotenone (1 μM)/antimycin A (1 μM) to compound 7-treated HCT116 cells. (B) Effect of compound 7 on intracellular oxygen tension. HCT116 cells were treated with compound 7 for 6 h under hypoxia condition. The intracellular oxygen tension was detected by hypoxia-sensitive probe MAR (0.5 μM). The scale bar indicates 200 μm. The fluorescence intensity of MAR probe was shown by vertical bars. Statistical significance: **P < 0.01, compared with hypoxia control.

Previously, we reported that MDH2 inhibition caused the suppression of mitochondrial respiration [14]. Therefore, we next examined the effects of compound 7 on the mitochondrial functions of HCT116 cells by measuring oxygen consumption rate using a XF analyzer. Compound 7 reduced oxygen consumption rate in a concentration-dependent manner, indicating inhibition of mitochondrial respiration (Fig 3A). In particular, compound 7 reduced the FCCP-induced maximal respiration rate of mitochondria. These results suggest that compound 7 suppresses mitochondrial respiration by inhibiting MDH2 activity in colorectal cancer cells. The reduction in oxygen consumption in the presence of compound 7 is expected to increase intracellular oxygen content. Therefore, we determined the oxygen level in the cells treated with compound 7 using a fluorescent hypoxia detection probe, mono azo rhodamine (MAR). In the compound 7-treated cells, the fluorescence of MAR was substantially decreased, indicative of increased oxygen content (Fig 3B), which may stimulate degradation of HIF-1α

### Compound 7 inhibits hypoxia-induced accumulation of HIF-1α

**Fig 4:**
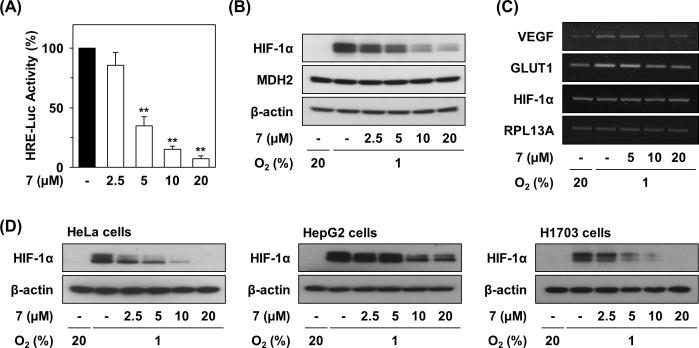
Inhibition of hypoxia-induced HIF-1α activation and tumor growth by compound 7. (A) Effect of compound 7 on HIF-1α activation. The hypoxia-induced transcriptional activation of HIF were determined by a cell-based HRE reporter gene assay. HCT116 cells stably expressing HRE-luciferase reporter gene were incubated for 12 h with or without drugs under normoxic or hypoxic conditions. After removing the supernatant, luciferase activity was measured. Statistical significance: **P < 0.01, compared with untreated control. (B) Effects of compound 7 on hypoxia-induced HIF-1α accumulation. The protein levels in compound 7-treated cells were detected by immunoblot analysis in HCT116 cells. (C) Effects of compound 7 on expression of mRNA level of HIF-1α target genes. The mRNA levels were detected by RT-PCR analysis. (D) Inhibitory effects of compound 7 on HIF-1α accumulation in various cancer cells.

We next examined the effects of compound 7 on the transcriptional activation of HIF-1α using HCT116 cells stably expressing HRE-luciferase [14]. As shown in Fig 4A, compound 7 inhibited the hypoxia-induced transcriptional activation of HIF-1α (IC_50_ = 3.58 μM). Furthermore, compound 7 reduced HIF-1α accumulation in a dose-dependent manner (Fig 4B). However, compound 7 did not affect MDH2 protein expression. When mRNA expressions of HIF-1α target genes were determined, compound 7 suppressed the mRNA levels of VEGF and GLUT1 but not HIF-1α (Fig 4C). This result suggests that HIF-1α is degraded at the post-transcriptional level via elevated intracellular oxygen caused by the inhibition of mitochondrial respiration, leading to reduced transcription of HIF-1α target genes during hypoxia.

We next examined whether Compound 7 reduced HIF-1α accumulation in various cancer cell lines. In the presence of Compound 7, HIF-1α protein levels were clearly decreased in HeLa, HepG2, and H1703 cells under hypoxic condition, indicating the similar effect of compound 7 in different types of cancer cells (Fig 4D).

### Compound 7 activates AMPK by reducing ATP production

**Fig 5:**
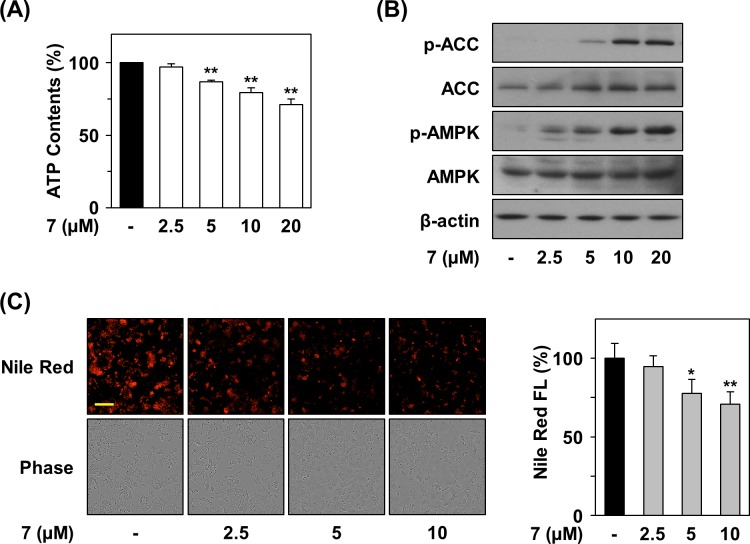
Reduction of intracellular ATP content and fatty acids by compound 7. (A) Effect of compound 7 on ATP level. After treatment of HCT116 cells with compound 7 for 6 hr, intracellular ATP content was measured using a luciferase-based assay system. (B) Activation of ATP-related signaling by compound 7. The protein levels in compound 7-treated cells were detected by immunoblot analysis. (C) Suppression of level of intracellular fatty acids by compound 7. HCT116 cells were incubated with compound 7 for 48 hr, and then intracellular fatty acids were stained with Nile Red. Statistical significance: *P < 0.05 and **P < 0.01, compared with untreated control.

We next confirmed that defects in mitochondrial respiration by compound 7 led to decreased ATP production (Fig 5A). An elevation in the ratio of AMP/ATP induces activation of AMP-activated protein kinase (AMPK) [21]; therefore, we examined the effect of compound 7 on the phosphorylation of AMPK in HCT116 cells (Fig 5B). Compound 7 increased the phosphorylation of AMPK, which suggests activation of AMPK downstream signaling. AMPK inhibits biosynthetic processes such as gluconeogenesis as well as lipid and protein synthesis by phosphorylation of a number of enzymes. Compound 7 treatment resulted in phosphorylation of acetyl-CoA carboxylase (ACC), a kinase involved in inhibiting fatty acid synthesis [22]. Moreover, Nile red staining revealed that compound 7 significantly reduced levels of fatty acids at concentrations that induced the phosphorylation of AMPK and ACC (Fig 5C).

**Fig 6:**
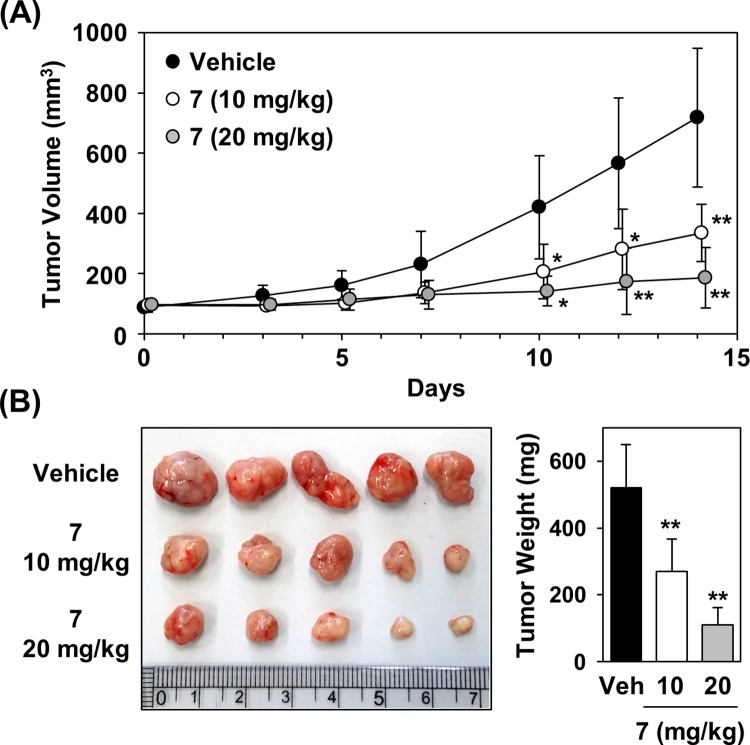
*In vivo* anti-tumor effects of compound 7 in HCT116 xenograft model. (A) Inhibition of tumor growth by compound 7. Total 5 × 10^6^ HCT116 cells were subcutaneously inoculated in the right flank of female nude mice. Drugs were administered intraperitoneally once a day for 14 days after reaching a tumor volume of 80–100 mm^3^. (B) Images of xenografts and tumor weight in control group and compound 7-treated group at the end of the experiment. Statistical significance: **P < 0.01, compared with vehicle control.

Next, the *in vivo* anti-tumor efficacy of compound 7 was analyzed by a xenograft assay in a HCT116 mouse model. Intraperitoneal administration of compound 7 daily resulted in significant efficacy on the tumor growth inhibition by 53% (10 mg/kg) and 74% (20 mg/kg) (Fig 6). Moreover, no significant adverse effects were observed in mice treated with compound 7.

### Discussion

The metabolic abnormalities of cancer that have recently been in the spotlight are caused by mitochondrial dysfunction [23,24]. The expression of various mitochondrial enzymes, such as citrate synthase [25], malic enzyme [26], malate dehydrogenase [17], aconitase [27], and succinate dehydrogenase [28], control cellular processes involved in tumor formation and cancer cell growth. Therefore, the enzymes involved in metabolic abnormalities in cancer are considered to be crucial targets for anticancer drug development.

We previously reported that LW6, a MDH2 inhibitor, suppressed HIF-1α accumulation [14]. In attempt to identify a compound targeting MDH2 for anti-cancer therapy, we performed virtual screening of a compound library. After determining MDH2 activities of 16 candidates, a benohydrazide based chemical, compound 7 was identified as a potent inhibitor of MDH2 activity and was selected for further study. Compound 7 inhibited MDH2 activity in a competitive fashion, similar to LW6. Due to the role of MDH2 in the TCA cycle in the mitochondrial matrix, we assessed whether compound 7 affects mitochondrial respiration in cancer cells. We determined that compound 7 reduced the oxygen consumption rate upon FCCP-induced maximal mitochondrial respiration in colorectal cancer cells. Use of a fluorescent hypoxia probe MAR revealed increased intracellular oxygen content in the presence compound 7, resulting in the degradation of HIF-1α. Furthermore, we confirmed that compound 7 inhibited the accumulation of HIF-1α in HCT116 cells.

On the other hand, ATP production was decreased due to defective mitochondrial respiration in the presence of compound 7, leading to the elevation of the AMP/ATP ratio. We determined that compound 7 treatment resulted in increased phosphorylation and activation of AMP-activated protein kinase (AMPK). AMPK negatively regulates ATP-consuming biosynthetic processes including gluconeogenesis as well as lipid and protein synthesis. AMPK phosphorylates of a number of enzymes directly involved in these processes and impacts transcriptional control of metabolism by phosphorylating transcription factors, co-activators, and co-repressors. Compound 7 treatment resulted in increased phosphorylation of acetyl-CoA carboxylase (ACC), a kinase involved in fatty acid synthesis that is negatively regulated by phosphorylation [22], indicating inhibition of fatty acid synthesis upon compound 7 treatment. We clearly demonstrated that compound 7 inhibited lipid accumulation inside cells by Nile Red staining. Furthermore, compound 7 significantly inhibited tumor growth determined by an *in vivo* xenograft assay in mice harboring HCT116 cells.

MDH2 has not been a popular cancer-related target of interest, unlike fumarate hydratase (FH), succinate dehydrogenase (SDH) and isocitrate dehydrogenase (IDH). The accumulation of succinate or fumarate by inactivating mutations in SDH or FH stabilizes HIF-1α through competitive inhibition of hydroxylation on prolyl hydroxylase domain (PHD) [29]. IDH mutations convert α-ketoglutarate (α-KG) to 2-hydroxyglutarate (2-HG), which can impair normal epigenetic regulation through inhibition of the TET2 family of DNA hydroxylases and the JmjC family of histone demethylases [30]. However, 2HG is suggested to promote degradation of HIF-1α [31].

Recently, several studies have reported cancer-associated functions of MDH2 [17,18]. Elevation of MDH2 expression has been implicated in docetaxel resistance in prostate cancer cells [17]. MDH2 knockdown using shRNA enhanced docetaxel sensitivity through activation of JNK signaling in prostate cancer cells. Similarly, a recent study also demonstrated that MDH2 is overexpressed in the mitochondrial fraction of doxorubicin-resistant uterine sarcoma cancer MES-SA cells [18]. The MDH2 knockdown of doxorubicin-resistant cells significantly potentiated doxorubicin-mediated cell growth inhibition. Therefore, inhibitors of MDH2 may be useful in mediating cancer cell resistance to chemotherapy. Moreover, a novel MDH2 inhibitor Compound 7 identified by virtual screening also demonstrated strong anti-tumor activity, suggesting MDH2 as a prospective therapeutic target of cancer.

We found that inhibition of MDH2 activity could lead to suppression of mitochondrial respiration. Because NADH is a cofactor in MDH2 enzyme reactions as an initial reactant and is required to generate protons in the electron transport chain (ETC), the inhibition of mitochondrial respiration by an MDH2 inhibitor might be mediated by the reduction of NADH.

This study presents several novel findings. First, a novel MDH2 inhibitor, compound 7, was identified as potential anti-cancer drug, which validates MDH2 as a promising therapeutic target of cancer. Second, we confirmed the mechanism of action of compound 7 in the suppression of HIF-1α accumulation by reducing oxygen consumption and ATP production. Third, compound 7 produced a significant impact on cancer metabolism that translated into anti-cancer efficacy in cancer cells.

## Conclusions

We identified compound 7, a novel MDH2 inhibitor, through ligand-based virtual screening. Compound 7 inhibited MDH2 activity via competitive fashion. Under hypoxic condition, Compound 7 reduced mitochondrial respiration and enhanced intracellular oxygen tension, thereby leading to degradation of HIF-1α. This study has provided clear evidence that MDH2 inhibitors exert anti-cancer activity, and compound 7 could be used to develop an anti-cancer drug to regulate cancer metabolism.
